# Sexual Dimorphism of Synaptic Plasticity Changes in CA1 Hippocampal Networks in Hypergravity-Exposed Mice—New Insights for Cognition in Space

**DOI:** 10.3390/cells14151186

**Published:** 2025-07-31

**Authors:** Mathilde Wullen, Valentine Bouet, Thomas Freret, Jean-Marie Billard

**Affiliations:** Campus Horowitz, Normandie Université, UNICAEN, INSERM, CYCERON, CHU Caen, COMETE UMR 1075, 14000 Caen, France

**Keywords:** hypergravity, electrophysiology, long term potentiation, NMDA receptor, long term depression, memory

## Abstract

Background: We recently reported sex-dependent impairment in cognitive functions in male and female mice exposed for 24 h, 48 h or 15 days to 2G hypergravity (HG). Methods: In the present study, we investigated brain functional correlates by analyzing synaptic activity and plasticity in the CA1 area of the hippocampus in both genders of mice previously exposed to 2G for the same duration. This was assessed by electrophysiological extracellular recordings in ex vivo slice preparations. Results: Basal synaptic transmission and glutamate release were unchanged regardless of HG duration. However, plasticity was altered in a sex- and time-specific manner. In males, long-term potentiation (LTP) induced by strong high-frequency stimulation and NMDA receptor (NMDAr) activation was reduced by 26% after 24 h of exposure but recovered at later timepoints. This deficit was reversed by D-serine or glycine, suggesting decreased activation at the NMDAr co-agonist site. In females, LTP deficits (23%) were found only after 15 days following mild theta burst stimulation and were not reversed by D-serine. Long-term depression (LTD) was unaffected in both sexes. Conclusions: This study highlights, for the first time, sex-dependent divergence in the CA1 hippocampal plasticity timeline following 2G exposure. The synaptic changes depend on exposure duration and the stimulation protocol and could underlie the previously observed cognitive deficits.

## 1. Introduction

Cognition, one of the brain’s most complex and integrated functions, relies on intricate neural dynamics shaped by numerous environmental inputs [1,2]. Among these, gravity has drawn increasing attention in recent decades due to the growing interest in space exploration [3]. Indeed, the pursuit of space travel means astronauts are exposed to varying gravitational conditions, alternating between microgravity (MG) and hypergravity (HG), which can potentially impact their performance by altering sensory input, motor control and cognitive function [4,5,6].

To date, the effects of gravitational changes on cognitive abilities remain poorly understood, even though some studies have observed structural changes in the brain [5,6,7]. To investigate how changes in gravitational force affect cognitive performance, preclinical research has mainly used specialized centrifuges to simulate HG conditions in small animals [8]. These rotating devices reproduce the gravitational shifts experienced during spaceflight and primarily stimulate the vestibular system, as well as sensorimotor and cutaneous inputs, all of which contribute to the central nervous system’s overall processing of gravity-related information [9,10,11,12,13].

Beyond balance and orientation, the vestibular system is also connected to brain regions involved in cognition, such as the hippocampus and prefrontal cortex [14,15,16]. As a result, disruptions in vestibular signals caused by gravitational changes could have widespread effects, potentially impairing cognitive functions by disrupting hippocampal networks.

Previous studies have reported some impacts of HG on brain functionality, particularly on hippocampal synaptic plasticity. For instance, Ishii et al. (2004) demonstrated that increased gravitational load (4G) can induce long-term potentiation (LTP) in the hippocampus of mice, suggesting that gravity may directly modulate synaptic efficacy [17]. Conversely, Lee et al. (2020) reported that chronic exposure to 4G HG impairs LTP in rats, as well as altering cognitive abilities [18]. While these results indicate that gravitational changes can influence synaptic plasticity within neuronal networks, they do not clearly determine whether this effect is positive or negative or the underlying mechanisms. Importantly, these studies did not consider whether these effects might differ between males and females. However, observations after spaceflights have already shown differential deterioration between men and women, notably in musculoskeletal and cardiac function but also in cognitive function [19,20,21,22,23,24]. In addition, we recently reported sexual dimorphism in the cognitive function of mice when experimenting with different patterns of 2G exposure.

Finally, only one recent study, to date, has considered the synaptic mechanisms that could be altered by HG in neuronal networks [18]. This question is, therefore, still an open issue, and the question of whether possible functional alterations could differ between sexes also remains to be addressed.

This study was, therefore, designed to investigate and compare, in male and female C57BL/6J mice, the changes in CA1 hippocampal neuronal network activity and plasticity induced by incremental time exposures (24 h, 48 h and 15 days) to moderate HG (2G). Furthermore, particular interest was focused on the synaptic deregulation mechanisms that could underlie HG effects in both genders of mice.

## 2. Materials and Methods

### 2.1. Animals and Hypergravity Exposure

Experiments were performed in accordance with the French and European Community guidelines for the care and use of laboratory animals (2010/63/EU) and approved under project number 2023041911573213 v4 (approval date 10 September 2023). For these experiments, a total of 120 adult (10–14-week-old) C57BL/6J mice were purchased from Janvier labs (Le Genest-Saint-Isle, 53940, France), including 71 males and 59 females. In addition, 10–14-week-old serine racemase knock-out (SR/KO) male mice (n = 10) were purchased from the Centre Universitaire de Ressources Biologiques (CURB, University of Normandie, Caen, France). Animals were kept in groups of 5 in a reversed 12 h/12 h light/dark cycle (light on at 7 p.m. and off at 7 a.m.), with regulated temperature (22 ± 1 °C) and humidity (55 ± 10%). The mice were randomly placed in cages using the Randmice site. Food and water were given ad libitum.

After a week of acclimatization, the mice were transferred to a centrifuge specially designed for small animals (CNES, National Center for Space Studies), which consists of four arms of 73 cm crossed around a central rotating axis [8]. Four free-swinging gondolas (55.5 cm × 38.5 cm × 31 cm) hung at the extremity of each arm. Each mouse cage (42 cm × 26 cm × 19 cm) was placed in a gondola with food and water ad libitum. The room was kept at the same temperature, humidity level and on a light/dark cycle as the accommodation room. 

The rotation speed was set to 45 r.p.m to obtain a gravito-inertial acceleration of 2G on the floor of the gondolas. Three different durations of HG exposure were applied: 24 h, 48 h and 15 days. The duration of exposure to HG was defined based on the literature to assess the period of habituation to the modified gravity environment (24 and 48 h) [17] and the time taken by the mice to become accustomed to their new environment (15 days) [18]. The centrifuge was stopped each day for less than 10 min in order to check and weigh the mice and clean the cages as necessary. Mice behavior was also monitored and controlled by cameras (Axis M1031-W, Axis communications^®^, Lund, Sweden) in the gondolas during the rotation. The device did not allow precise analysis of behavior but allowed a minimal visual check of animal welfare. Control (1G) animals were housed in similar gondolas as the centrifuged mice. Gondolas were placed in the room of the centrifuge, so that mice were exposed to the same olfactory and auditory stimuli as 2G animals.

### 2.2. Brain Slice Preparation

At the end of the HG exposure, mice were taken out from the centrifuge and immediately anesthetized with 5% isoflurane and decapitated. Each brain was rapidly removed from its skull and placed 1 min in chilled (−3 to 0 °C) artificial cerebrospinal fluid (aCSF) containing 124 mM NaCl, 3.5 mM KCl, 1.5 mM MgSO_4_, 2.3 mM CaCl_2_, 26.2 mM NaHCO_3_, 1.2 mM NaH_2_PO_4_ and 11 mM glucose (pH 7.4). Transverse slices (400 µm thick) of the whole hippocampus were obtained using a slicer (Mc Ilwain^®^, Hemmant, Australia) and placed in aCSF gassed with a 95%O_2_/5%CO_2_ mixture in a holding chamber maintained at 28 °C for at least 1 h. One slice was then transferred to a submersion-type recording chamber, where it was maintained between two nylon meshes and continuously perfused at room temperature with the same gassed aCSF. Non-blind extracellular recordings were obtained using glass micropipettes (2–5 MΩ) filled with 2 M NaCl positioned in the stratum radiatum of the CA1 hippocampal area.

All pharmacological agents were diluted directly in the perfusion medium from stock solutions prepared in distilled water. All drugs were purchased from Tocris Bio-science^®^ (Bristol, UK).

### 2.3. Electrophysiological Measurements

#### 2.3.1. Basal Synaptic Transmission

To assess basal synaptic transmission, presynaptic fiber volleys (PFVs) and non-N-methyl-D-aspartate receptor (non-NMDAr)-mediated field excitatory postsynaptic potentials (fEPSPs) were evoked at 0.1 Hz by electrical stimulation of Schaffer collaterals and commissural fibers located in the *stratum radiatum*. The averaged slope of three successive PFVs and fEPSPs was measured using WinLTP^©^ 3.00 software [25]. To quantify the level of synaptic activation, an index of synaptic efficacy (Ise) corresponding to the fEPSP/PFV ratio was calculated and plotted against stimulus intensity (300, 400 and 500 μA).

#### 2.3.2. Paired-Pulse Facilitation

A paradigm of paired-pulse facilitation (PPF) was then induced to investigate possible changes in the presynaptic mechanisms of glutamate release, using paired pulses with inter-stimulus intervals of 30 ms. The level of PPF was determined as the ratio of the slope of the second response over that of the first fEPSP.

#### 2.3.3. NMDA Receptor Activation

As LTP expression is closely linked to activation of the NMDA subtype of glutamate receptors [26,27,28], the specific synaptic potentials mediated by these receptors were investigated. Briefly, specific NMDAr-mediated fEPSPs were pharmacologically isolated from slices perfused with aCSF containing low Mg^2+^ (0.1 mM) supplemented with the non-NMDAr antagonist 2,3-dioxo-6-nitro-1,2,3,4-tetrahydrobenzoquinoxaline-7-sulfonamide (NBQX, 10 µM). One salient feature of NMDAr activation is the requirement for the binding of the endogenous co-agonists D-serine or glycine in addition to glutamate for channel opening. Consequently, the responsiveness of NMDAr-mediated fEPSPs was assessed at saturating doses of the amino acid D-serine (defined as 100 μM in [29]) and glycine (defined as 500 μM in [30]). These saturating doses of the co-agonists thus enable all synaptic NMDArs to be recruited by a single test stimulation in slices from both control and HG-exposed mice. The effects were assessed by comparing the Ise calculated before and 15 min after the addition of the co-agonist to the aCSF.

#### 2.3.4. Functional Plasticity

Plasticity of synaptic strength is a critical process for learning and memory [31,32,33]. Therefore, changes in synaptic plasticity have been investigated using different paradigms of tetanus stimulation to induce either long-term potentiation or depression.

In order to investigate long-term potentiation (LTP) of synaptic transmission, a test stimulus was applied every 10 s and adjusted to obtain an fEPSP with a baseline slope of 0.1 V/s. The averaged slope of three fEPSPs was measured during 15 min for a baseline before the delivery of a high-frequency stimulation (HFS) consisting of one burst at 100 Hz pulses delivered for 1 s. In another set of experiments, a theta burst stimulation (TBS) was delivered, consisting of 4 successive sequences delivered at 0.1 Hz. Each sequence consisted of 5 bursts of 4 impulses delivered at 100 Hz and separated from each other by 200 ms (5 Hz). Using these different stimulation protocols is useful to consider possible changes in the excitation/inhibition balance [34].

In addition, long-term depression (LTD) of synaptic transmission was investigated after a low-frequency (2 Hz) conditioning stimulation was applied for 10 min after the baseline. This was interesting to investigate possible HG-dependent alterations of the metabotropic properties of the NMDAr, which has recently been proposed to govern LTD expression [35]. In this case, the test stimulation was adjusted to obtain an fEPSP with a baseline slope of 0.2 V/s.

In both LTP (HFS and TBS) and LTD experiments, testing with a single pulse was then resumed for 60 min to determine the level of synaptic potentiation/depression, which was averaged from the last 15 min of recording, e.g., between 45 and 60 min after the conditioning stimulation. In recordings performed in the presence of D-serine or glycine, the co-agonist was applied 10 min before initiating the baseline and maintained throughout the recording.

### 2.4. Data Analysis

Normality of data distribution and homogeneity of variances were assessed to define the statistical tests. If data followed a normal distribution and variances were homogeneous, an intergroup comparison was performed using ANOVA and Dunnett’s method for post hoc analysis. Comparisons with a reference value or between two groups were performed using paired or unpaired sample *t*-tests. When data were not normally distributed, intergroup comparisons were performed using the Kruskal–Wallis test, followed by the Bonferroni–Dunn post hoc test for multiple comparisons. Statistical analyses were performed using GraphPad Prism 8.0, and *p*-values less than 0.05 were considered significant (GraphPad^®^ Software, San Diego, CA, USA).

## 3. Results

### 3.1. Basal Synaptic Transmission

**Figure 1:**
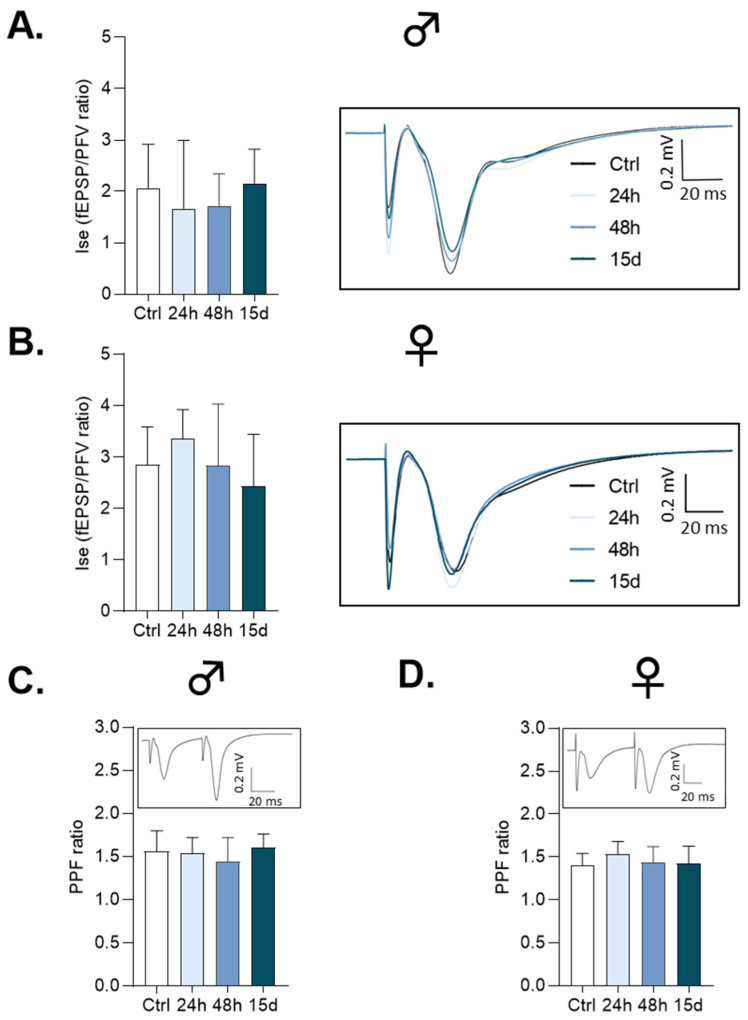
HG does not affect basal functional synaptic activity in CA1 hippocampal networks. For all graphs, data are represented as Mean ± SEM. Inserts show superimposed representative traces obtained in respective experimental groups. (**A**) Index of synaptic efficacy (Ise) obtained with 400 μA stimulation intensity in slices from male mice. (**B**) Ise obtained with 400 μA stimulation intensity in slices from female mice. (**C**) Paired-pulse facilitation (PPF) ratio in slices from male mice. (**D**) PPF ratio in slices from female mice.

The effects of HG exposure on basal synaptic transmission mediated by non-NMDA glutamate receptors at CA3/CA1 synapses were first assessed by changes in the fEPSP/PFV ratio, which is considered an index of synaptic efficacy (Ise). In both males and females, the Ise was not statistically different between control and HG-exposed mice, regardless of the exposure duration (Figure 1A: control (n = 29), HG-exposed for 24 h (n = 30), 48 h (n = 30) and 15 days (n = 30), *Anova test*, F3, 115 = 1.25, *p* > 0.05 and Figure 1B: control (n = 31), HG-exposed for 24 h (n = 32), 48 h (n = 30) and 15 days (n = 33), *Anova test*, F3, 122 = 2.06, *p* > 0.05). Profiles were obtained, regardless of the intensity of the stimulation used to activate the afferent fibers (300, 400 or 500 μA). Additionally, no difference between male and female mice was found when the control and respective HG-exposed groups were compared.

### 3.2. Paired-Pulse Facilitation

The absence of HG-related effects on basal synaptic transmission suggests that the synaptic mechanisms governing glutamate release are not affected by gravitational changes. To further support this postulate, paired stimuli were applied, which is an electrophysiological paradigm classically used to estimate alterations in the presynaptic release of the excitatory neurotransmitter. Accordingly, delivering two stimuli at 30 ms intervals induced facilitation of the second fEPSP (Figure 1C,D, inserts), which allowed the PPF ratio to be calculated. As illustrated, the PPF ratio was not statistically modified between control and HG-exposed male or female mice, regardless of the duration of HG exposure (Figure 1C: control (n = 46), HG-exposed for 24 h (n = 48), 48 h (n = 49) and 15 days (n = 48), *Kruskal–Wallis test*, *p* > 0.05 (n = 48) and Figure 1D: control (n = 51), HG-exposed for 24 h (n = 51), 48 h (n = 50) and 15 days (n = 54), *Anova test*, F3, 201 = 2.62, *p* > 0.05). Furthermore, no sex-dependent difference was shown within each experimental group.

### 3.3. Functional Plasticity

**Figure 2:**
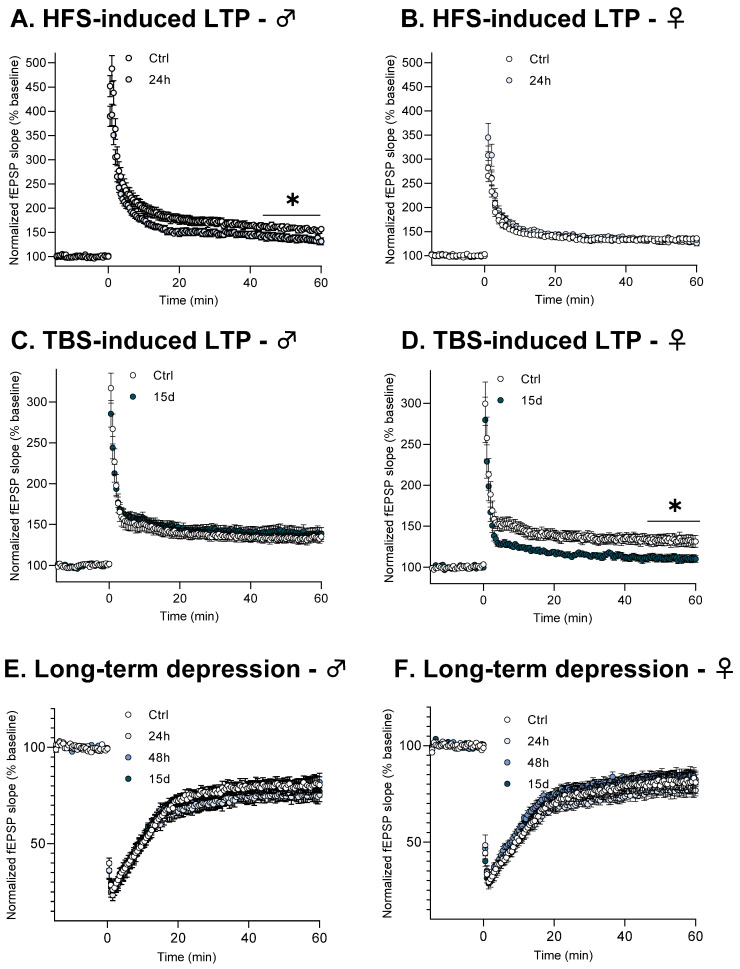
HG differentially affects functional synaptic plasticity of CA1 hippocampal networks according to sex and conditioning stimulation. For all graphs, data are represented as Mean ± SEM. *: *p* < 0.05. (**A**) Time-course of long-term potentiation (LTP) induced by 1 × 100 Hz tetanus (high-frequency stimulation, HFS) in slices from control males and males exposed to HG for 24 h. (**B**) Time-course of HFS-induced LTP in slices from control female mice and female mice exposed to HG for 24 h. (**C**) Time-course of LTP induced by theta burst stimulation (TBS) in slices from control male mice and male mice exposed to HG for 15 days. (**D**) Time-course of TBS-induced LTP in slices from control female mice and female mice exposed to HG for 15 days. (**E**) Time-course of long-term depression (LTD) induced in slices from control and HG-exposed male mice. (**F**) Time-course of LTD induced in slices from control and HG-exposed female mice.

High frequency-related conditioning stimulation (HFS) induced robust and long-term potentiation (LTP) of synaptic strength in all animal groups (Figure 2A: control (n = 19), HG-exposed for 24 h (n = 19), 48 h (n = 18) and 15 days (n = 18) and Figure 2B: control (n = 20), HG-exposed for 24 h (n = 20), 48 h (n = 20) and 15 days (n = 20), *Anova test*, F3, 76 = 0.28, *p* > 0.05). Indeed, the mean fEPSP slope over the last 15 min of recordings was significantly higher than that averaged from the baseline (see Supplementary Data Figure S1A,B to view LTP magnitude for all groups). However, male mice exposed to a short (24 h) period of HG displayed decreased LTP. Indeed, LTP magnitude was statistically reduced in male mice exposed to HG for 24 h (*32.1 ± 5.9*% of baseline) when compared to male controls (*58 ± 5.2*% of baseline, *p* = 0.03, Kruskal–Wallis test with Bonferroni–Dunn post hoc test).

Similarly, theta burst stimulation (TBS) induced LTP in all experimental groups (Figure 2C: control (n = 18), HG-exposed for 24 h (n = 15), 48 h (n = 20) and 15 days (n = 15)*, Kruskal–Wallis test*, *p* > 0.05 and Figure 2D: control (n = 15), HG-exposed for 24 h (n = 15), 48 h (n = 14) and 15 days (n = 14)). No deficit was observed in male mice, regardless of the duration of HG exposure (see Supplementary Data Figure S1C,D to view LTP magnitude for all groups). Conversely, with TBS stimulation, a significant deficit was observed in female mice after a long duration (15 days) of HG exposure (*p* = 0.03, Kruskal–Wallis test with Bonferroni–Dunn post hoc test). When compared to the respective control group (*32.6 ± 6.5*% of baseline), the LTP magnitude of female mice with 15 days of HG exposure showed a significant decrease (*9.7 ± 2.8*%).

On the other hand, LTD was also induced in all experimental groups by a low-frequency conditioning stimulation; however, with this conditional stimulation no significant alteration was found regardless of sex and exposure duration to HG (Figure 2E: control (n = 12), HG-exposed for 24 h (n = 12), 48 h (n = 12) and 15 days (n = 12), *Anova test*, F3, 44 = 0.35, *p* > 0.05 and Figure 2F: control (n = 12), HG-exposed for 24 h (n = 12), 48 h (n = 12) and 15 days (n = 12), *Anova test*, F3, 44 = 0.62, *p* > 0.05) (see Supplementary Data Figure S1E,F to view LTD magnitude for all groups).

Taken together, these results indicate that HG impacts LTP magnitude depending on sex, strength of the conditioning stimulation and duration of exposure but does not affect LTD expression. As a next step, we investigated the synaptic mechanisms that could underlie the HG-related sexual dimorphism of LTP alterations.

### 3.4. NMDA Receptor Activation

**Figure 3:**
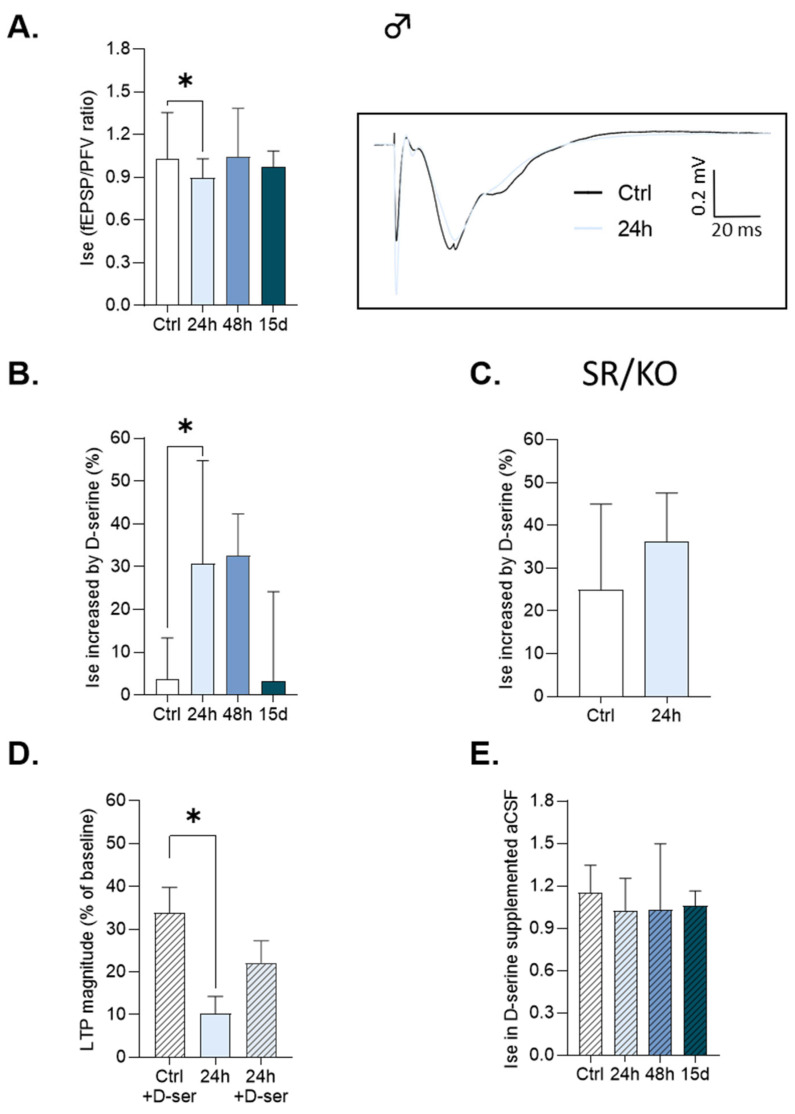
D-serine mediates the decrease in HFS-induced LTP in male mice exposed to HG for 24 h. For all graphs, data are represented as Mean ± SEM. *: *p* < 0.05. (**A**) Index of synaptic efficacy (I_se_) obtained with 400 μA stimulation intensity of specific NMDAR fEPSPs in slices from male mice. The insert shows the superimposed respective traces of isolated NMDAR fEPSPs obtained in a control and a male mouse exposed to HG for 24 h. (**B**) The percentage of the Ise increases after the addition of D-serine (100 μM) in control and HG-exposed male mice. (**C**) The percentage of the Ise increases after the addition of D-serine (100 μM) in control and serine racemase knock-out (SR/KO) male mice exposed to HG for 24 h. (**D**) The LTP magnitude obtained in slices from male mice after perfusion with D-serine (100 μM). (**E**) The Ise calculated for 400 μA stimulation intensity of specific NMDAR fEPSPs in slices from control and HG-exposed male mice after the addition of D-serine (100 μM).

**Figure 4:**
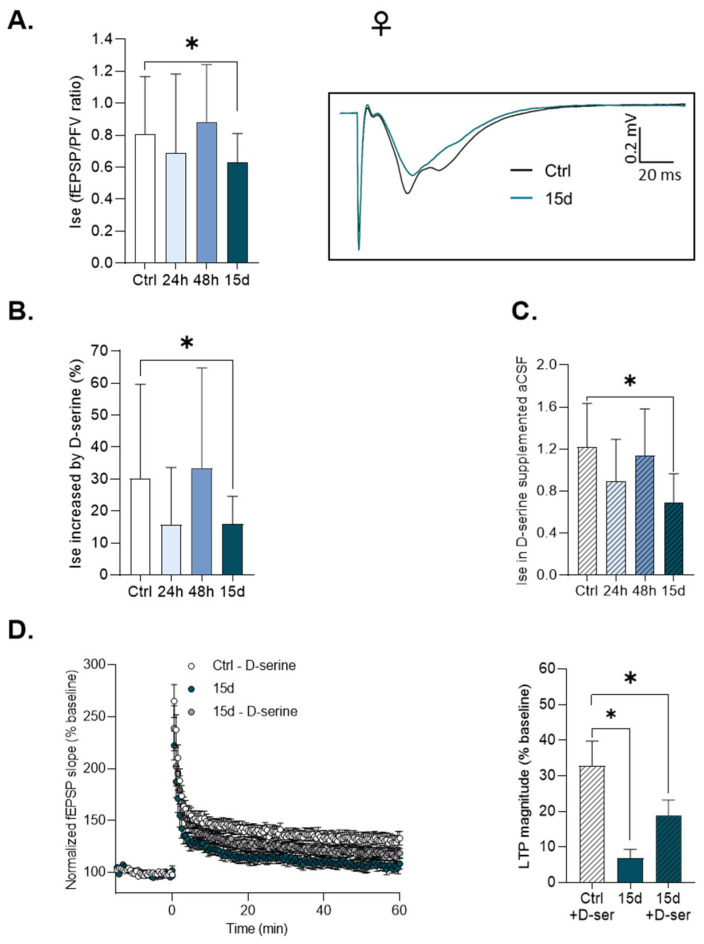
D-serine does not mediate the decrease in TBS-induced LTP in female mice exposed to HG for 15 days. For all graphs, data are represented as Mean ± SEM. *: *p* < 0.05. (**A**) The index of synaptic efficacy (I_se_) calculated for 400 μA stimulation intensity of isolated NMDAR fEPSPs in slices from female mice. The insert shows the superimposed respective traces of isolated NMDAR fEPSPs obtained in control female mice and female mice exposed to HG for 15 d. (**B**) The percentage of the Ise increases after the addition of D-serine (100 μM) in control and HG-exposed female mice. (**C**) The I_se_ was calculated for 400 μA stimulation intensity of isolated NMDAR fEPSPs in slices from control and HG-exposed female mice after the addition of D-serine (100 μM). (**D**) Left: The time-course of TBS-induced LTP in slices from control female mice and female mice exposed to HG for 15 days after perfusion with D-serine (100 μM). Right: LTP magnitude obtained in slices from female mice after perfusion with D-serine (100 μM).

Long-lasting fEPSPs, specifically due to NMDAr activation, were recorded (Figure 3A and Figure 4A, inserts). Interestingly, sex-dependent alterations were found, closely matching LTP deficits and emphasizing the critical role of NMDAr in the HG-dependent effects on synaptic plasticity.

Indeed, the Ise was significantly reduced after 24 h of HG exposure in male mice, but not after 48 h or 15 days (Figure 3A), compared to control animals (*p* = 0.03, unpaired *t*-test, control (n = 14), HG-exposed for 24 h (n = 14), 48 h (n = 15) and 15 d (n = 13)). At least two mechanisms could account for this weaker NMDAr-mediated synaptic activity in males exposed for 24 h, including a decrease in receptor density and/or impaired mechanisms of activation.

As illustrated in Figure 3B, an increase in the NMDAr-related Ise induced by the bath application of D-serine was significantly higher in male mice exposed to HG for 24 h (*D-serine: 32.3 ± 7.9*% vs. *controls: 3.8 ± 4.6*% calculated for 400 μA stimulation intensity, *p* = 0.04, Kruskal–Wallis test with Bonferroni–Dunn post hoc test), but not for 48 h or 15 days, compared to controls. A similar increase in the NMDAr-related Ise was obtained after 24 h of HG exposure with glycine application (*glycine: 31.1 ± 7.4*% vs. *controls: 18.28 ± 4.4*%).

Such a higher responsiveness to exogenous co-agonists only in mice exposed for 24 h strongly suggests changes in the occupancy of the NMDAr co-agonist binding sites at this delay of 2G exposure.

To further assess this synaptic deregulation, we then exposed serine racemase knockout (SRKO) mice to 2G for 24 h [36]. These animals have a complete deletion of exon 1 of the SR, reducing cerebral D-serine levels by at least 90%, which is compensated by higher levels of glycine. In these exposed KO mice, the responsiveness of NMDAr-mediated fEPSPs to the exogenous application of D-serine was not exacerbated (*D-serine: 35.1 ± 5.2*% vs. *controls: 27.7± 5.9%, unpaired t-test*, Figure 3C). This result, therefore, indicates that the lower occupancy of NMDAr co-agonist binding sites recorded in control male mice exposed for 24 h is specifically related to changes in the synaptic availability of D-serine. Accordingly, we found that the decrease in LTP expression displayed by control male mice exposed to HG for 24 h was rescued after supplementing slices with D-serine (*Kruskal–Wallis test with Bonferroni–Dunn post hoc test, controls with D-serine: 33.76 ± 6*% vs. *24 h: 10.27 ± 3.98*, *p* = 0.01 and control with D-serine vs. *24 h with D-serine: 22.16 ± 5.1%, p* > *0.05*) (Figure 3D: control (n = 14) and HG-exposed for 24 h (n = 13)), confirming the significant contribution of this amino acid in the deficit of synaptic plasticity associated with 24 h exposure to 2G.

Regarding possible changes in NMDAr density, it is worth noting that the NMDAr-related Ise was comparable in control and mice exposed for 24 h when slices were supplemented by exogenous D-serine or glycine and when the maximal recruitment of synaptic receptors was obtained (Figure 3E: *Anova test*, F3, 52 = 1.76, *p* > 0.05). This result, therefore, indicates that NMDAr density per se is not modified by HG exposure for 24 h.

A different profile of changes in NMDAr activation by HG was found in female mice since the Ise decreased after 15 days but not after 24 h or 48 h of exposure (Figure 4A: *p* = 0.04, unpaired *t*-test, control (n = 16), HG-exposed for 24 h (n = 16), 48 h (n = 16) and 15 d (n = 17)). In addition, a striking difference in male mice with regard to the exogenous application of the co-agonist was noted. Indeed, in 15-day-exposed female mice with lowered NMDAr activation, the increase due to exogenous D-serine (Figure 4B) was significantly weaker compared to control mice (*D-serine: 13.8 ± 5.4*% vs. *controls: 35.4 ± 7.0*%, *p* = 0.01 ANOVA with Dunnett’s post hoc test). This is in contrast to altered NMDAr activation in male mice exposed for 24 h, in which the responsiveness to the exogenous application of the co-agonist was exacerbated (Figure 3B). In addition, the Ise determined in the presence of saturating D-serine remained significantly decreased in female mice with 15 days of HG exposure compared to control mice (Figure 4C: *unpaired t-test*, Ctrl compared to 15 d, *p* = 0.005), and delivering the co-agonist to slices of those animals failed to prevent the TBS-induced LTP deficit (Figure 4D: control (n = 19) and HG-exposed for 24 h (n = 16), *Kruskal–Wallis test and Dunn’s multiple comparisons test*, *p* = 0.0001). All together, these results suggest that the late depressive effect of HG exposure on TBS-induced LTP and NMDAr activation in female mice involves a decrease in receptor density rather than weaker synaptic availability of the co-agonist.

## 4. Discussion

This electrophysiological study aimed to investigate the effects of increased periods of 2G exposure on CA1 hippocampal synaptic activity and plasticity. In addition, experiments were performed on male and female mice, and the sex-dependent effect was investigated. Regardless of the sex of animals, basal synaptic transmission and glutamate release were not impacted by HG. In contrast, NMDAr activation and the related expression of synaptic plasticity were altered depending not only on the sex of animals but also on the duration of exposure to HG and the type of conditioning stimulation. In addition, this study provides new data regarding sex-related differences in synaptic mechanisms underlying the effects of HG on NMDAr-dependent functional plasticity in CA1 hippocampal networks.

Among the multiple challenges facing the ability to travel into space, ensuring optimal permanent control of cognitive abilities, which are essential for the success of missions, is paramount [37]. We know that changes in gravity, such as HG, stimulate the vestibular system. The vestibular system projects to various brain regions and may thus influence multiple physiological functions, including cardiovascular regulation, motor control and cognition [3,12,38,39,40]. Studies conducted on murine models that have used different gravity changes (2G or 4G) have demonstrated alterations in memory and spatial navigation associated with the hippocampus [18,41]. In consideration of the established indirect connections between the vestibular system and the hippocampus [4,14,42], HG exposure is expected to have an impact on hippocampal functioning, but this needs to be precisely defined to enable safe space missions.

So far, only a few animal studies have examined the effects of HG exposure on hippocampal synaptic activity and plasticity. To date, it has been suggested that an increase in the gravity field could induce changes in neuronal networks, but this would require relatively high (4G) and prolonged (>48 h) exposure. In fact, Ishii et al. (2004) showed that 48 h of exposure to 4G, but not 2G, could enhance LTP in the hippocampus of mice [17]. Conversely, Lee et al. (2020) found that exposure to the same level of HG for four weeks impaired LTP in rats [18]. However, it is important to note that the exposure times and species used in the respective studies were not the same. In addition, no significant change was described after shorter exposure. In accordance with the literature, we confirm in this paper that basal neurotransmission in hippocampal networks is not affected by 2G stimulation, regardless of the duration of HG exposure [43]. Conversely, impairment of hippocampal plasticity (CA1 LTP) is observed with different sensitivity according to sex and/or the duration of exposure, as well as the nature of the conditioning stimulus. Thus, males displayed a deficit in HFS-induced LTP after only a short duration of HG exposure (24 h). In addition, female mice showed a TBS-induced LTP deficit that needed a long duration (15 days) of HG exposure. As pointed out above, Ishii and colleagues (2004) reported that 2G exposure for 24 h did not elicit a deficit of CA1 hippocampal synaptic plasticity in male C57Bl6 mice [17]. This discrepancy with our results may possibly be related to the respective designed procedures. Indeed, functional plasticity was assessed in our study immediately after the completion of centrifugation, while electrophysiological measurements were performed more than 24 h after centrifugation in Ishii’s study, which may have permitted full recovery in that case.

While our study shows that the impairment of LTP expression by 2G exposure relies on impaired activation of the NMDA subtype of glutamate receptors, it also provides, for the first time, functional evidence of sexual dimorphism regarding the synaptic mechanisms underlying this impaired NMDAr activation by HG.

On the one hand, weaker occupancy of the co-agonist binding sites, which is critical for NMDAr activation [44,45], occurs only in male mice, which could rely on decreased synaptic availability of the endogenous co-agonist D-serine. This amino acid is synthesized from the precursor L-serine by serine racemase [46] and released in the brain through a D-serine shuttle involving a dual dialogue between neurons and surrounding astrocytes [47,48]. Attenuation of astrocyte reactivity is induced by 2G exposure [49] and could, therefore, affect the functionality of the D-serine shuttle. Alternately, a significant amount of D-serine is produced by intestinal bacteria and delivered by systemic circulation to the brain across the blood–brain barrier (BBB) [50]. In addition, diffusion of L-serine across the BBB by the Slc38a5 transporter also significantly contributes to brain D-serine levels [51]. Interestingly, impairment of BBB permeability after 24 h of 2G exposure has recently been characterized in male mice [52,53], which, therefore, closely correlates with the period of weaker synaptic availability of D-serine found in the present study. These mechanisms could, therefore, also be considered possible alternatives involved in impaired NMDAr activation. In addition, our results suggest that both altered astrocyte reactivity and BBB impairment could be rapidly compensated, since the altered NMDAr activation by D-serine is not found after longer periods of HG exposure, but this possibility remains to be definitively investigated. Regardless of the underlying mechanism, lower occupancy of the NMDAr co-agonist binding sites in male mice exposed for 24 h could impact the magnitude of HFS-induced LTP because this form of synaptic plasticity requires a much higher pool of active NMDAr than TBS-induced LTP to be generated.

On the other hand, the hypothesis of a decrease in co-agonist binding site occupancy to account for the impaired NMDAr activation in female mice after 15 days’ exposure to HG is unlikely because the deficit was not alleviated by saturating levels of D-serine at synapses to recruit all NMDAr [29]. In female mice, a decrease in NMDAr density may be hypothesized, and the question of whether this loss of synaptic receptors is irreversible remains to be addressed. This decrease could also explain why TBS-induced and not HFS-induced LTP is preferentially affected, considering the lower threshold for this form of functional plasticity to be generated.

Regarding LTD expression, this form of functional synaptic plasticity was initially considered to result from weak and delayed NMDAr-dependent ionotropic signaling in response to the low frequency and long-lasting duration of the conditioning stimulation [54,55]. However, it is currently accepted that LTD is driven by conformational changes of the NMDAr [35]. This metabotropic effect of the receptor has been identified in numerous processes, both in neurons and non-neuronal cell types, and has been involved in the physiopathology of several neurological diseases [56]. Our results, therefore, indicate that in contrast to LTP, LTD expression was not affected by HG, regardless of sex and time exposure, suggesting that this form of metabotropic signaling is not sensitive to gravity changes, at least until 2G.

This study, therefore, emphasizes sexual dimorphism regarding the effects of HG on NMDAr-dependent synaptic plasticity, a dimorphism that we have also recently observed in behavioral investigations. Indeed, we observed that spatial working and object recognition-related memories are differentially altered in male and female mice receiving similar protocols of 2G exposure, as delivered in the present functional study. Although a direct correlation between electrophysiological and behavioral approaches is not possible, given that the cohorts were not the same, a comparison does not reveal a clear-cut overlap between changes in synaptic plasticity and memory deficits, regardless of sex or time exposure. One has to keep in mind that the present electrophysiological analysis was limited to the CA1 hippocampal area. A more extensive functional analysis, including the CA3 field and dentate gyrus or possibly other cerebral structures involved in cognition and vestibular regulation, would, therefore, be essential to better consider possible correlations. In addition, since ex vivo electrophysiological studies exhibit individual variability, a correlation at the level of the same mice would be advantageous.

## 5. Conclusions

There are still many avenues to explore further regarding the mechanisms underlying 2G-related alterations of CA1 hippocampal functional plasticity and to extend our knowledge to the entire hippocampus. For example, hippocampal assays of endogenous D-serine or NMDAr subunit expression could provide additional insights, as well as determination of the status of sex hormones to investigate sexual dimorphism. Furthermore, it would be important to assess plasticity recovery over time once the mice have returned to 1G gravity. There is no doubt that these future directions will refine our understanding of how different sexes are vulnerable to environmental stressors, such as HG. Although it is currently quite ambitious to extrapolate our preclinical results to humans, we aim that, in the future, they could be of particular relevance in the search for sex-specific interventions related to space missions.

## Data Availability

Data are available by request from the author for correspondence.

## Supplementary Materials

The following supporting information can be downloaded at: https://www.mdpi.com/article/10.3390/cells14151186/s1, Figure S1. HG differentially affects functional synaptic plasticity of CA1 hippocampal networks according to sex and conditioning stimulation. For all graph, data are represented as Mean ± SEM, *: *p* < 0.05. A. HFS LTP magnitude determined for the last 15min of recording in slices from male mice (Kruskal-Wallis test with Bonferroni–Dunn post hoc tests, *p* = 0.03). B. HFS LTP magnitude obtained in slices from female mice (Anova test, F3, 76 = 0.28, *p* > 0.05). C. TBS LTP magnitude obtained in slices from male mice (Kruskal-Wallis test, *p* > 0.05). D. TBS LTP magnitude obtained in slices from female mice (Kruskal-wallis test with Bonferroni–Dunn post hoc test, *p* = 0.03). E. LTD magnitude determined in slices from male mice (Anova test, F3, 44 = 0.35, *p* > 0.05). F. LTD magnitude in slices from female mice (Anova test, F3, 44 = 0.62, *p* > 0.05).

## Abbreviations

The following abbreviations are used in this manuscript:

aCSF	Artificial cerebrospinal fluid
AMPA	α-amino-3-hydroxy-5methyl-4-isoxazolepropionic acid
AMPAr	α-amino-3-hydroxy-5methyl-4-isoxazolepropionic acid receptor
BBB	Blood–brain barrier
CNES	French National Center for Space Studies
CURB	Biological Resources University Center
HFS	High-frequency stimulation
HG	Hypergravity
ISE	Index of synaptic efficacy
LTD	Long-term depression
LTP	Long-term potentiation
MG	Microgravity
NMDA	N-methyl-D-aspartate
NMDAr	N-methyl-D-aspartate receptor
PFVs	Presynaptic fiber volleys
RPM	Revolution per minute
SR/KO	Serine racemase knock-out
TBS	Theta burst stimulation
