# Supplementary material for: Sexual Dimorphism of Synaptic Plasticity Changes in CA1 Hippocampal Networks in Hypergravity-Exposed Mice—New Insights for Cognition in Space
Source: Cells. 2025 Jul 31;14(15):1186. doi: 10.3390/cells14151186 (PMC12346747; doi:10.3390/cells14151186)
Supplement: Supplementary file 1 [file cells-14-01186-s001.zip › cells-3728054-supplementary.pdf]

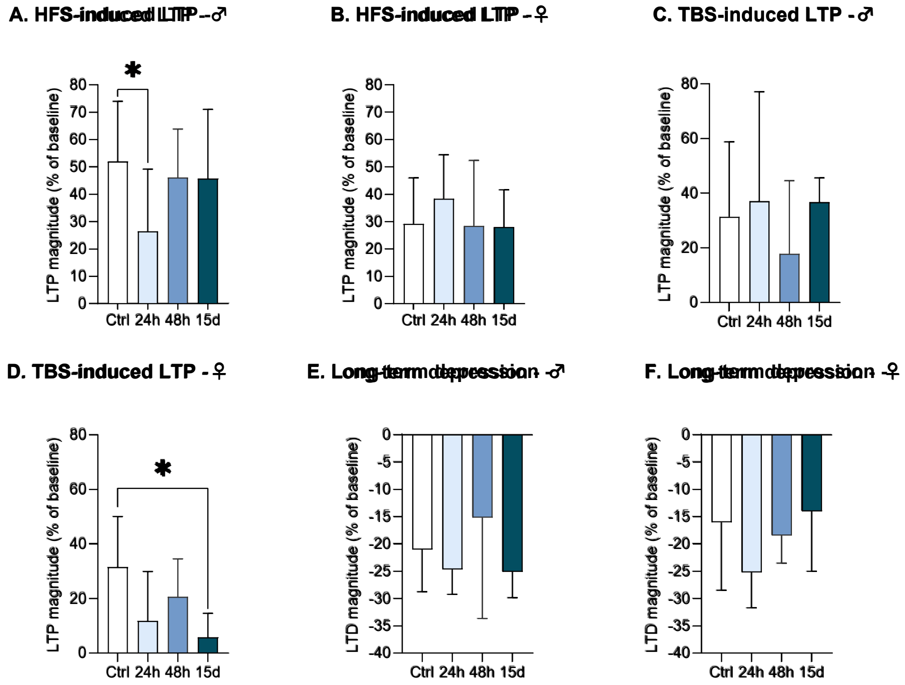

Supplementary data Figure S1. HG differentially affects functional synaptic plasticity of CA1 hippocampal networks according to sex and conditioning stimulation. For all graph, data are represented as Mean±SEM, *: p<0.05. **A**. HFS LTP magnitude determined for the last 15min of recording in slices from male mice *(Kruskal-Wallis test with Bonferroni–Dunn post hoc tests,* p = 0.03). **B**. HFS LTP magnitude obtained in slices from female mice (*Anova test,* F3, 76 = 0.28, p > 0.05). **C**. TBS LTP magnitude obtained in slices from male mice *(Kruskal-Wallis test,* p > 0.05). **D**. TBS LTP magnitude obtained in slices from female mice (*Kruskal-wallis test with Bonferroni–Dunn post hoc test,* p = 0.03). **E**. LTD magnitude determined in slices from male mice (*Anova test,* F3, 44 = 0.35, p > 0.05). **F**. LTD magnitude in slices from female mice (*Anova test,* F3, 44 = 0.62, p > 0.05).
